# Improving Nitrogen Efficiency: Lessons from Malawi and Michigan

**DOI:** 10.1100/tsw.2001.307

**Published:** 2001-11-09

**Authors:** Sieglinde Snapp, Heather Borden, David Rohrbach

**Affiliations:** Department of Horticulture, Michigan State University, East Lansing 48824, USA

**Keywords:** nutrient budgeting, farm surveys, potato and maize systems, participatory research, nitrogen management and modeling

## Abstract

Two case studies are presented here of nitrogen (N) dynamics in potato/maize systems. Contrasting systems were investigated from (1) the highland tropics of Dedza, Malawi in southern Africa and (2) the northern temperate Great Lakes region of Michigan. Formal surveys were conducted to document grower perceptions and N management strategies. Survey data were linked with N budgets conducted by reviewing on-farm data from representative farms in the targeted agroecosystems and simulation modeling to estimate N losses. Potential N-loss junctures were identified. Interventions that farmers might accept are discussed. The Malawi system uses targeted application of very small amounts of fertilizer (average 18 kg N ha) to growing plants. This low rate is on the steep part of plant response to N curve and should serve to enhance efficiency; plant growth, however, is generally stunted in Malawi due to degraded soils and weed competition. Very limited crop yields reduce N efficiency from a simulated 60 kg grain per kg N to an actual of ~20 kg grain per kg N (at 40 kg N ha applied). Legume-intensified systems could improve growth potential and restore N use efficiency through amelioration of soil quality and transfer functions and from biological fixation N inputs. In the Michigan system, N efficiency is enhanced currently through multiple, split applications of N fertilizer tailored to plant growth rate and demand. Fertilizer N rates used by growers, however, averaged 32% higher than recommended rates and 40% higher than N removed in crop product. Application of 50 kg N ha to cover crops in the fall may contribute to the apparent high potential for N leaching losses. Careful consideration of N credits from legumes and residual soil N would improve N efficiency. Overall, N budgets indicated 0 to 20 kg N ha loss potential from the Malawi systems and tenfold higher loss potential from current practice in Michigan maize/potato rotations. Best management practices, with or without integration of legumes, could potentially reduce N losses in Michigan to a more acceptable level of about 40 kg N ha.

## INTRODUCTION

Motivations vary, but improving N efficiency is a primary goal of farmers, scientists, and policy makers in temperate and tropical agroecosystems. Efficient N use addresses the need to limit environmental risks from nonpoint source water pollution in the

Northern Hemisphere. Simultaneously it addresses the challenge of widespread nutrient deficiencies in Sub-Saharan Africa and other developing economies. N efficiency is defined in widely disparate terms and at different scales, from grain yield produced per N fertilizer used to plant uptake of N per soil N supply to photosynthesis per leaf N[1]. In this paper we discuss N efficiency as a quantifiable measure of productivity per unit measure of N supplied.

Leached nitrate from agricultural systems is becoming a contaminant of surface and groundwater. It is one of the most widespread, nonpoint source water pollutants in the U.S.[2]. Potato (*Solanum tuberosum* L.) production systems have high potential for nitrate leaching[3]. This is not surprising since irrigated sandy loam soils are widely used to produce potatoes, and potatoes are one of the most N-inefficient crop plants. For example an average of only 33% of applied N was recovered by a potato crop in a Minnesota study[4]. Cereal grain crops generally average higher N efficiency in the range of 35 to 55%[1].

Malawi cropping systems are constrained by a unimodal precipitation pattern that limits farmers to one crop per season and to an average area of land per farm holding of less than 2 ha[9] . Soils are Alfisols and Ultisols, generally sandy in texture and deficient in N status. Maize (*Zea mays*) accounts for 60 to 80% of the area sown. The remainder of smallholder arable land is sown to tobacco (*Nicotiana tabacum*), groundnut (*Arachis hypogaea*), common bean (*Phaseolus vulgaris*), pigeonpea (*Cajanus cajan*), and, in highland sites, potato (*Solanum tuberosum*)[9]. Fertilizer is frequently applied 2 to 4 weeks after crop emergence. This may be a risk avoidance strategy of resource-poor farmers to target fertilizers to established plants[10].

Michigan potato production is concentrated on sandy soils underlain by abundant supplies of accessible irrigation water. Total potato area has averaged about 20,000 ha over the state for the last 5 years. The majority of producers rotate potatoes with maize and occasionally with a legume crop such as green bean[11]; 2-year rotations are used on about 55% of the hectarage, whereas 3- or 4-year rotations are common in lower population density areas. Farm size is highly variable, from 5 ha fresh market producers to 1000-plus ha farms producing primarily for potato snack processors. Michigan growers generally use multiple split applications of N fertilizer to improve synchrony of N supply and crop demand. However residual soil N levels appear to be high, indicating excess N is applied.

Agroecosystem analysis can be used to identify and explore critical intervention points in N management practices[5]. For example, N budgets have documented that N loss from legume sources is surprisingly low in organic field crop systems[6]. At a larger scale, village- and regional-level nutrient balances and modeling have elucidated nutrient efficiency in Sub-Saharan Africa[7,8]. In the study reported here, we use simulation to evaluate simple input/output N budgets of maize/potato rotations in Michigan and maize/potato intercrop systems in Malawi. The objectives were to document farmer perceptions, N management practice, and simulation of N budgets for typical maize and potato production systems. The systems studied were representative of contrasting agroecologies and highlighted potential N loss points and opportunities for improving efficiency[3].

## EXPERIMENTAL METHODS

### Farm Surveys

The Malawi farm survey was conducted in June of 1999 using a questionnaire administered to 70 randomly selected farmers in Dedza, a high altitude region of central Malawi. The survey collected data on farm resources, fertility management strategies, cropping systems, crop choice, and N management practice. Interviews were conducted with the head of household, the spouse, or both key decision makers when possible.

The U.S. farm survey was conducted in collaboration with the Michigan Potato Industry Commission in March 2001. The questionnaire was mailed to all commercial potato growers in Michigan. Follow-up telephone calls were conducted to encourage growers to complete and return questionnaires. The 35 growers that returned the survey represent 40% of the potato production area. The survey collected data on farm resources, cropping systems, N management, pest problems, and pest management practices. Questions related to N strategies included cover crops, N monitoring tools used, N fertilizer practice, and N application decision-making criteria.

Data entry and analysis were conducted using the Microsoft Excel data spreadsheet and the statistical package Statistica[12]. Descriptive statistics of the variables were calculated. Open-ended questions were evaluated by determining the major categories represented by the answers and then calculating the percentage of responses per category.

### Nitrogen Budgets

Malawi crop rotation productivity and N data were modeled based on average cropping system population densities and soil characteristics from field-monitoring exercises conducted in conjunction with trials carried out in Dedza, the location of the farm survey (14°20'S 34°30'E, 1600 m asl). Soil samples from the top 0 to 25 cm were collected from 40 fields at the end of the growing season in 1998. Ten subsamples were mixed for a composite representative sample at each site. Samples were air dried for at least 96 h, ground to pass through a 2-mm sieve, and analyzed for nitrate in a 2M potassium chloride extract and organic carbon by acid dichromate digestion[14,15]. Soil pH was read in a suspension of 1:2.5 soil:distilled water. Texture was determined using a hydrometer in a dispersant solution of 3% sodium hexametaphosphate. Soil organic carbon averaged 12 g $kg^{-1}$ across the sites and was lower than 8 g $kg^{-1}$ on very sandy soils. The soil pH (1:2.5 soil/water ratio) varied from moderately to slightly acid. These soil characteristics are consistent with previous observations from central Malawi[16].

The crop and soil simulation model used in Malawi was the Agricultural Production Systems Simulator (APSIM) . This model has been tested extensively in the semi-arid tropics, including a decade of on-farm verification in Kenya and 2 years evaluation in Malawi[1,17,18]. Three cropping systems were compared: (1) current practice, which is a maize continuous system using the average fertilizer rate of 18 kg N $ha^{-1}$ from survey data (lowdensity potato intercrop at a 6:1 ratio of maize:potato was not possible to simulate given current model limitations), (2) recommended practice, which is a maize continuous system at the recommended rate of 69 kg N $ha^{-1}$, over threefold higher than current fertilizer use, and (3) integrated practice, which is rotation of a grain legume with maize; the recommended N fertilizer rate for the system in the second year is 69 kg N $ha^{-1}$ minus legume N credit of 35 kg N $ha^{-1}$ from residues incorporated after grain legume rotation. Pigeonpea was the grain legume of choice since it consistently has been shown to perform well under low soil fertility on smallholder farms and to produce residues containing ~60 kg N $ha^{-1}$ where we assume from N mineralization assays that 35 kg N $ha^{-1}$ is available to subsequent crops[9,10]. This is similar to results from other Malawi studies of on-farm production of pigeonpea residues, although considerably less than is biologically possible[13,15]. We used a grain legume rotation scenario as incorporation of a cover crop or green manure would jeopardize food security on small landholdings and is not widely adoptable[9]. The Malawi government–recommended fertilizer rate was developed through country-wide trials that targeted recommendations by agroecozone and yield response[19].

Michigan crop rotation productivity and associated N budgets are adapted from a source that modeled with coefficients based on expert opinion and survey data, using Decision Support System for Agrotechnology Transfer (DSSAT) 3.0 software[3,22]. Three cropping systems were compared (Table 2): (1) current practice, which is a maize/rye winter cover crop/potato rotation sequence (average N fertilizer rates in kg N $ha^{-1}$ from survey data: 210 for maize, 290 for potatoes, and 50 for cover crops), (2) recommended practice, which is a maize/rye winter cover crop/potato rotation sequence with recommended N fertilizer rates (approximately 90 kg N $ha^{-1}$ lower than current practice), and (3) integrated practice, which is a maize/legume winter cover crop/potato rotation sequence (recommended N fertilizer rate minus 2-year accumulated legume N credit = 116 kg N $ha^{-1}$, Michigan recommendation used to estimate 45% available = 52 kg N $ha^{-1}$)[20]. Hairy vetch residue N accumulated as a winter cover crop is estimated from biomass measurements on-farm. Two-year scenarios were used to compare systems.

## RESULTS AND DISCUSSION

### Survey Results

Malawi smallholder farmers use fertilizer sparingly if at all. In our survey an average of 18 kg N $ha^{-1}$ was applied to a cash crop of maize intercropped with potato and use has decreased in the last 5 years (Table 1). This may be driven in part by a marked devaluation of local currency, reduced government subsidies, and an escalating fertilizer price. Between 1990 and 1996, the maize grain to fertilizer price ratio declined by over 50%[19]. There is also evidence that many Malawian farmers no longer use fertilizers except on cash crops such as potato[9]. The greatest constraint to fertilizer use by the Malawi farmers surveyed was the expense involved (Table 1). Availability of fertilizers was also a problem. These results are not surprising since Malawi is consistently ranked among the poorest countries in the world. Farmers that have a very limited amount of cash to invest in fertilizer may prioritize highly efficient use of that fertilizer rather than optimizing biological performance[23]. This is not always reflected in current Malawi Ministry of Agriculture–recommended levels of fertilizer use, which target high yield goals[24].

In contrast to Malawi, N fertilizer use by the surveyed Michigan growers has remained relatively constant in recent years at around 290 kg N $ha^{-1}$ (Table 1). A surprising observation was the rate of fertilizer (50 kg N $ha^{-1}$) generally applied to winter cereal cover crops (generally rye, *Secale cereale*) at planting after potato harvest. Informal interviews suggest that growers apply this fertilizer as an insurance factor. The perception is that N fertilizer is required to ensure rapid establishment of the winter cover crop before cold temperatures halt growth. Yet on-farm trials in Michigan conducted over 3 years by Vitosh and colleagues[20] showed that residual soil inorganic-N after potato harvest is about 100 kg N $ha^{-1}$. Lowering fertilizer rates from an average of 300 to 210 kg N $ha^{-1}$ reduced potentially leachable N by 40%. No significant reduction in quality or quantity of potato and maize yield was observed with more moderate, recommended N fertilizer rates[20]. Vitosh's findings were communicated widely to growers in Michigan yet only 20% of growers surveyed used lower fertilizer rates. Furthermore, approximately 25% of the growers perceived their ability to use enough N as being curtailed by concerns about fertilizer expense, vine management, or nitrate leaching (Table 1). Washington State potato producers also perceived recommended N fertilizer levels as risky and used about one-third higher fertilizer rates than recommended[21].

Michigan growers are concerned about optimizing N management. This is indicated by the 75% of growers that regularly monitor N in plant and soil. However, farmers were highly risk-adverse and frequently maintained high levels of N fertilizer application that do not take into account soil N or legume-N credits. Almost one-third of growers have concerns about the reliability of N monitoring tools, such as potato petiole nitrate-N measurements (Table 1). Refining and validating these tools to improve grower confidence may be a key intervention to improve N efficiency.

### Nitrogen Budgets and Consequences for N Use Efficiency

Input and output budgets illustrate where excess nutrients might accumulate and be vulnerable to losses from leaching, erosion, or volatilization. We used survey data to calibrate three scenarios for each country, including current practice, recommended practice, and an integrated system that included a legume. Through simulation modeling, we estimated yield, crop product N, and N leaching for the different systems, and we estimated net N, the crop product N output subtracted from inputs, N fertilizer and legume N (Table 2) . This is an estimate of excess N that could be lost if it is not incorporated into soil organic matter or recycled by root uptake of subsequent crops.

Excess N was almost nil in the Malawi case (Table 2). This was due to low levels of N inputs and outputs and high N efficiency. Both simulation data and measured data from trials in Malawi show that N efficiency (kg grain per kg N applied) is

**TABLE 1**
**Nitrogen Management Survey Response Data from Producers of Maize and Potatoes in Dedza, Malawi and Michigan (including percentage of growers that gave the indicated response)**

| | Malawi (n = 70) | Michigan (n = 35) |
|---|---|---|
| **Grower Opinion** | % | |
| *N monitoring tools:* | | |
| Useful and reliable | na[a] | 42.9 |
| Useful but unreliable | na | 28.6 |
| Not useful and unreliable | na | 2.9 |
| Do not use | 100 | 25.6 |
| *Usage of N fertilizer now vs. 5 years ago:* | | |
| More | 4.3 | 12.5 |
| Same | 35.7 | 65.6 |
| Less | 60.0 | 21.9 |
| *Ability to use N fertilizer:* | | |
| Usually apply enough | 5.7 | 75.8 |
| Sometimes apply enough | 17.1 | 18.2 |
| Rarely apply enough | 77.2 | 6.0 |
| *Grower reasons for limiting N fertilizer use:* | | |
| Fertilizer cost | 92.9[b] | 44.4 |
| Fertilizer availability | 21.4 | 0 |
| Late blight/other diseases | 0 | 11.1 |
| Vine kill problems | 0 | 44.4 |
| Potential nitrogen leaching | 0 | 63.0 |
| Do not need fertilizer | 5.7 | 0 |
| **Grower Fertilizer Use[c]** | kg N ha$^{-1}$ | |
| Malawi maize/potato intercrop | 18 | na |
| Michigan potato + (winter cover crop, rye) | na | 290 (50) |
| Michigan maize | na | 210 |

[a] na = Not applicable.
[b] Growers could list more than one answer to this question so total is greater than 100%.
[c] Self-reported. Actual fertilizer use will vary; reports suggest that higher rates may be used in the U.S., particularly in high precipitation years[20,21].

relatively high for the small quantities of N fertilizer used (Figure 1). Legumes can also improve efficiency and may be a critical N source for farmers who cannot afford fertilizer. In Malawi a rotation with a legume grain crop reduced “excess” N by decreasing fertilizer inputs and maintained productivity at levels achievable with 69 kg N ha$^{-1}$ from fertilizer. This is moderately higher than on-farm trial results from maize/grain legume rotations[10]. The simulated N leaching was about 30 kg N ha$^{-1}$ for all systems (Table 2), similar to levels reported elsewhere[1]. Our on-farm soil nitrate-N measurements support this analysis with 0 to 25 kg N ha$^{-1}$ measured in the top 25 cm of soil (data not shown). Similar values of 0 to 18 kg N ha$^{-1}$ were measured at different landscape positions in southern Malawi[15].

Michigan soil-N and yields simulated were high, often more than sixfold higher than in Malawi. This is to be expected from a high-input, irrigated system compared to resource-constrained smallholder production. High productivity potential and low N efficiency of irrigated production systems are associated with losses of ~70%[25]. Raun and Johnson[25] suggest major changes in agricultural practice are necessary to improve the efficiency of irrigated systems; yet, we found that following recommended practices significantly reduced simulated N leaching (Table 2). Legume cover crops and use of a legume N credit, as well as recommended fertilizer rates, were sufficient to reduce net N to about 40 kg N ha$^{-1}$ compared to 220 kg N ha$^{-1}$ associated with current practice (Table 2). To put this in perspective, 40 kg N ha$^{-1}$ has been suggested as an

**TABLE 2**
**Simulated Crop Yield and N Data from Malawi and Michigan, Comparing Three Different Systems**

| | | Malawi | | | Michigan | | |
|---|---|---|---|---|---|---|---|
| **Model Predictions:** | **Year** | **Current Practice** | **Recommended N** | **Integrated System** | **Current Practice** | **Recommended N** | **Integrated System** |
| Grain legume yield | y1 | 0 | 0 | 1135 | 0 | 0 | 0 |
| Maize yield[a] | y1 | 1215 | 3033 | 0 | 9460 | 9191 | 8976 |
| *N fertilizer added* | y1 | 18 | 69 | 0 | 210 | 160 | 133 |
| Crop product N removed | y1 | 22 | 52 | 27 | 189 | 184 | 180 |
| N leached | y1 | 27 | 37 | 29 | 91 | 73 | 38 |
| Cover crop residue N | y1 and y2 | 0 | 0 | 0 | 0 | 0 | 116 |
| *N credit from legume* (kg ha$^{-1}$) | y1 and y2 | 0 | 0 | (35) | 0 | 0 | (52) |
| Maize yield[b] | y2 | 1620 | 3874 | 3752 | 0 | 0 | 0 |
| Potato yield (fresh wt.) | y2 | nd[c] | nd | nd | 44,800 | 43,700 | 45,400 |
| *N fertilizer added* | y2 | 18 | 69 | 35 | 290 | 200 | 173 |
| *Crop product N removed* | y2 | 29 | 66 | 64 | 132 | 129 | 134 |
| N leached | y2 | 30 | 42 | 34 | 146 | 110 | 60 |
| N fertilizer cover crop | y2 | 0 | 0 | 0 | 50 | 10 | 0 |
| ***Nitrogen budget:*** | | | | | | | |
| N fertilizer added + legume N credit | Over 2 years | 36 | 138 | 70 | 540 | 370 | 358 |
| Crop product N removed | Over 2 years | 51 | 117 | 91 | 333 | 324 | 325 |
| ***NET Nitrogen*** | **Over 2 years** | **–15** | **21** | **–21** | **217** | **46** | **33** |

*Note:* N budgets calculated as inputs (fertilizer and legume credit) minus outputs (N removed in crop product), based on simulation data.

[a] Maize yields simulated for year 1 in Malawi are average yields from 10-year simulation with APSIM using low rainfall years (precipitation data from years with less than 800 mm); Michigan simulated maize yield one is from data source[3], where DSSAT simulation was used with a nonlimiting water assumption, appropriate for this irrigated system.

[b] Maize yields simulated for year 2 in Malawi are average yields from 10-year simulation with APSIM using high rainfall years (precipitation data from years with over 800 mm).

[c] No data are shown as current simulation models do not adequately predict yields of potato in a low population density intercrop.

allowable leaching limit to protect against excess groundwater nitrate loading[3].

## CONCLUSIONS

Potential key points for N losses were identified. Eliminating N fertilizer application to winter cover crops and using recommended rates of N fertilizer would markedly reduce N losses in Michigan maize/potato systems. On the other hand, Malawi scenarios involved very low productivity levels with limited N inputs. Some gains in efficiency may be achievable through integration of legumes into the system but increasing overall yield levels and thus the potential for N response is critical. Current recommended practices in both the African and the U.S. systems were effective at reducing potential N loss. This simulation derived data need to be verified through more intensive on-farm monitoring; yet it appears that reasonably N efficient recommendations may already be in place. Farmer perceptions as well as the market price and policy context, however, may preclude adoption of these recommendations.

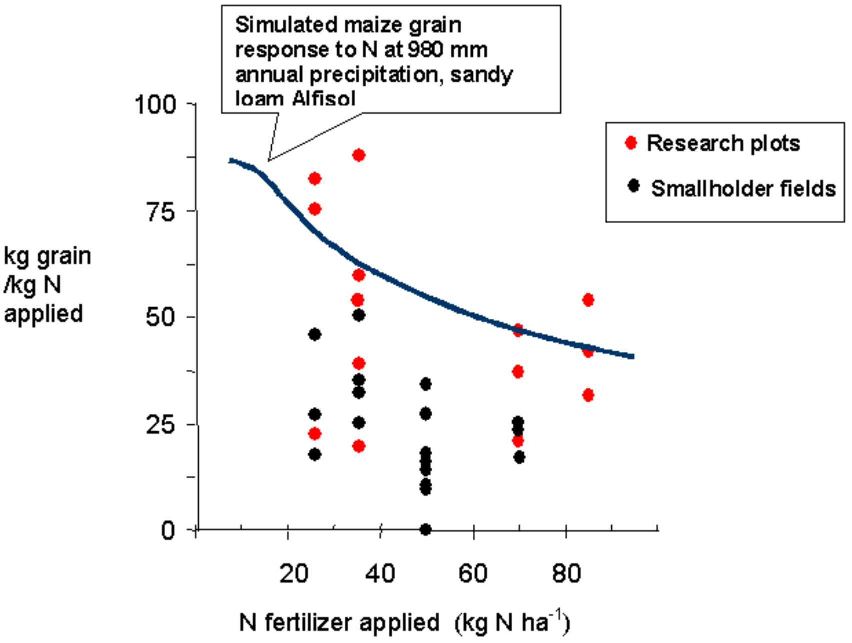

**FIGURE 1**. Maize grain yield response to N on a sandy loam low fertility soil. Simulated grain N efficiency (kg grain producer per N fertilizer applied) by APSIM is compared to actual values from field trials conducted under researcher or farmer management.

| | | |
|---|---|---|
| Received: | July | 18, 2001 |
| Revised: | October | 8, 2001 |
| Accepted: | October | 15, 2001 |
| Published: | November | 9, 2001 |

## BIOSKETCH

**Dr. Sieglinde (Sieg) Snapp** is Assistant Professor of vegetable integrated crop management in the Departments of Horticulture and Crop and Soil Sciences, Michigan State University. As a cropping systems scientist, he has 15 years of experience working on maize, bean, potato, and vegetable systems in Columbia, southern Africa, California, and most recently Michigan. From 1993 to 1999, Dr. Snapp worked with national scientists in southern Africa researching legume-intensified systems and integrated use of organic and inorganic nutrients. He developed farmer participatory research methods, e.g., the 'mother/baby trial' on-farm trial design with lattice square analysis, now used widely. His research interests include system design to improve nutrient efficiency, root system vigor, and soil quality.
